# Statement on chronotherapy for the treatment of hypertension: consensus document from the Korean society of hypertension

**DOI:** 10.1186/s40885-023-00249-2

**Published:** 2023-09-01

**Authors:** Sungha Park, Sang-Hyun Ihm, In-Jeong Cho, Dae-Hee Kim, Jae Hyeong Park, Woo-Baek Chung, Seonghoon Choi, Hae Young Lee, Hyeon Chang Kim, Il Suk Sohn, Eun Mi Lee, Ju Han Kim, Kwang-il Kim, Eun Joo Cho, Ki-Chul Sung, Jinho Shin, Wook Bum Pyun

**Affiliations:** 1 Division of Cardiology, Severance Cardiovascular Hospital, Integrative Research Center for Cerebrovascular and Cardiovascular Diseases, Yonsei University College of Medicine, Seoul, Republic of Korea; 2 Division of cardiology, Department of Internal Medicine, Bucheon St. Mary’s Hospital, College of Medicine, The Catholic University of Korea, Bucheon, Republic of Korea & Catholic Research Institute for Intractable Cardiovascular Disease, College of Medicine, The Catholic University of Korea, Seoul, Republic of Korea; 3 Division of Cardiology, Department of Internal Medicine, Ewha Womans University Seoul Hospital, Ewha Womans University College of Medicine, Seoul, Korea; 4 Division of Cardiology, Asan Medical Center, University of Ulsan College of Medicine, Seoul, Republic of Korea; 5 Department of Cardiology in Internal Medicine, Chungnam National University Hospital, Chungnam National University School of Medicine, Daejeon, Korea; 6 Division of Cardiology, Department of Internal Medicine, Seoul St Mary’s Hospital, The Catholic University, Seoul, South Korea; 7 Cardiology, Department of Internal Medicine, Kangnam Sacred Heart Hospital, Hallym University College of Medicine, Seoul, South Korea; 8 Division of Cardiology, Department of Internal Medicine, Seoul National University Hospital, Seoul National University College of Medicine, Seoul, South Korea; 9 Department of Preventive Medicine, Yonsei University College of Medicine, Seoul, Republic of Korea; 10 Department of Cardiology, Kyung Hee University Hospital at Gangdong, College of Medicine, Kyung Hee University, Seoul, Republic of Korea; 11 Division of Cardiology, Department of Internal Medicine, Wonkwang University Sanbon Hospital, Gunpo, Gyeonggi-do Republic of Korea; 12 Department of Cardiology, Chonnam National University Hospital, Gwangju, Korea; 13 Department of Internal Medicine, Director of Geriatric center, Seoul National University College of Medicine, Seoul National University Bundang Hospital, Seongnam-Si, Gyeonggi-Do Korea; 14 Division of Cardiology, Department of Internal Medicine, Yeouido St. Mary’s Hospital, The Catholic University College of Medicine, Seoul, Korea; 15 Division of Cardiology, Department of Internal Medicine, Kangbuk Samsung Hospital, Sungkyunkwan University School of Medicine, Seoul, Republic of Korea; 16 Division of Cardiology, Department of Internal Medicine, Hanyang University Seoul Hospital, Seoul, Korea

**Keywords:** Blood pressure, Hypertension, Chronotherapy, Antihypertensive

## Abstract

Nocturnal blood pressure (BP) has been shown to have a significant predictive value for cardiovascular disease. In some cases, it has a superior predictive value for future cardiovascular outcomes than daytime BP. As efficacy of BP medications wanes during nighttime and early morning, control of nocturnal hypertension and morning hypertension can be difficult. As such, chronotherapy, the dosing of BP medication in the evening, has been an ongoing topic of interest in the field of hypertension. Some studies have shown that chronotherapy is effective in reducing nocturnal BP, improving non dipping and rising patterns to dipping patterns, and improving cardiovascular prognosis. However, criticism and concerns have been raised regarding the design of these studies, such as the Hygia study, and the implausible clinical benefits in cardiovascular outcomes considering the degree of BP lowering from bedtime dosing. Studies have shown that there is no consistent evidence to suggest that routine administration of antihypertensive medications at bedtime can improve nocturnal BP and early morning BP control. However, in some cases of uncontrolled nocturnal hypertension and morning hypertension, such as in those with diabetes mellitus, chronic kidney disease, and obstructive sleep apnea, bedtime dosing has shown efficacy in reducing evening and early morning BP. The recently published the Treatment in Morning versus Evening (TIME) study failed to demonstrate benefit of bedtime dosing in reducing cardiovascular outcomes in patients with hypertension. With issues of the Hygia study and negative results from the TIME study, it is unclear at this time whether routine bedtime dosing is beneficial for reducing cardiovascular outcomes.

## Introduction

Studies have shown that ambulatory blood pressure monitoring (ABPM) is superior to clinic blood pressure (BP) in predicting cardiovascular outcomes [1]. Among parameters that can be measured by ABPM, nocturnal BP has been shown to have a significant predictive value for cardiovascular diseases. In some cases, it has a superior predictive value for future cardiovascular outcomes to daytime BP [2, 3]. Abnormalities in night time BP fall, namely non dippers and risers, have also been shown to be associated with adverse cardiovascular prognosis [2, 3]. As efficacy of BP medications wanes during night time and early morning, control of nocturnal hypertension and morning hypertension can be difficult. As such, chronotherapy, the dosing of BP medication in the evening/bedtime, has been an ongoing topic of interest in the field of hypertension. The possibility of chronotherapy being an effective treatment for nocturnal BP reduction was initially reported by Svensson et al. [4]. They analyzed 38 patients enrolled in the HOPE-trial who also underwent ABPM. Because the protocol of the HOPE-trial specified administration of Ramipril at night, investigators were able to analyze the efficacy of BP medication at bedtime compared to placebo. Their results demonstrated a significant, 17/8 mmHg lowering of nocturnal BP reduction for Ramipril without significant reduction in daytime BP, suggesting that chronotherapy might be an effective treatment for controlling nocturnal BP [4]. Subsequent studies, mainly from a single study group from Spain, have shown that chronotherapy is effective in reducing nocturnal BP, improving non dipping and riser patterns to dipping patterns, and improving cardiovascular prognosis [5–7]. However, criticism and concerns have been raised regarding the design of these studies and implausible clinical benefits in cardiovascular outcomes considering the degree of BP lowering from bedtime dosing [8–11]. The recently published The Treatment in Morning versus Evening (TIME) study also demonstrated a lack of benefit of evening dosing compared to morning dosing in terms of major cardiovascular outcomes in patients with hypertension [12]. Against the backdrop of these circumstances, the Korean Society of Hypertension (KSH) has developed a consensus document with regard to the role of chronotherapy in the management of hypertension.

## Chronobiology and nocturnal hypertension

Diurnal variation in BP is known to be controlled by multiple endogenous and exogenous factors [9]. Circadian control of the cardiovascular system is known to be controlled by a master pacemaker located in the suprachiasmatic nucleus (SCN) of the hypothalamus [13]. Cyclic, oscillometric expression of the core clock genes, the main helix-loop-helix transcription factors, circadian locomotor output cycles kaput (CLOCK), and brain and muscle ARNT (aryl hydrocarbon receptor nuclear translocator)-like protein-1 (BMAL1) in the SCN are important regulators of this circadian control [13]. A diurnal variation in WNT/β signaling that regulates GSK-3β phosphorylation in SCN astrocytes is also important in diurnal regulation of BP [14]. Dysregulation in the secretion of other neuropeptides such as vasopression, vasoactive intestinal polypeptide, and neurotensin, and dysregulated secretion of melatonin might also contribute to abnormalities in diurnal variation of BP [15, 16]. Aging is associated with impairment of baroreceptor function that contributes to higher BP variability [17]. Autonomic function has been shown to be important in regulating diurnal variation of BP as nocturnal BP with reverse dipping hypertension is associated with an increase in sympathetic nervous system activation [9, 18]. Lastly, salt sensitivity, sleep disturbance, and occupational day/night shift might also contribute to the disturbance of diurnal BP variation [9, 19]. As a consequence, BP at night may decrease by less than 10% (non dipping) or increase compared to daytime BP (Risers). To reduce the increase in night time BP, chronotherapy, or the administration of anti-hypertensive medications at night or bedtime, has been an ongoing topic of interest in the field of hypertension.

### Evidence for the efficacy of evening dosing in lowering nocturnal blood pressure

The efficacy of chronotherapy in reducing nocturnal BP has been presented mainly by the aforementioned Spain research group. In the MAPEC study by Hermida et al., 2156 hypertensive subjects were randomized to either morning dosing or ≥ 1 medications taken at bedtime [5]. That study showed that bedtime dosing resulted in a significantly greater reduction in asleep SBP compared to morning dosing (-11.8 ± 13.2 vs. -6.6 ± 12.5 mmHg, *P* < 0.001) [5]. In the Hygia Chronotherapy trial also performed by the Hermida group, bedtime dosing of ≥ 1 medications was associated with significantly higher percentage of nighttime BP lowering (12.2 ± 7.7 vs. 8.5 ± 8.4%, *P* < 0.001) than morning dosing [7]. However, results from previous studies regarding chronotherapy were inconsistent. In a meta-analysis of 21 randomized controlled trials in 1,993 patients with primary hypertension, there was no significant difference between bedtime dosing and morning dosing for reduction of morning BP [20]. In a multicenter, randomized, double blind trial that randomized valsartan 320 mg to AM or PM dosing, there was no significant benefit of PM dosing in terms of nocturnal BP or early morning BP reduction [21]. A randomized crossover trial of 103 patients with relatively well controlled hypertension on ≥ 1 antihypertensive medications also showed that morning and bedtime dosing had no significant difference in daytime or night time BP lowering [22]. The above mentioned findings might be explained by the fact that while medications with short half-lives might not sufficiently lower nocturnal or early morning BP [23], the timing of administration would not matter for the majority of antihypertensive drugs with sufficient half-lives once they are in a steady state [24]. However, as bedtime dosing could allow for peak effect in the early morning, theoretically it should have better efficacy in lowering nocturnal BP in those with non-dipping patterns and nocturnal hypertension. Some hypertensive patients are known to have high percentage of nocturnal hypertension and non-dipping status, such as those with diabetes mellitus (DM), chronic kidney disease (CKD), and obstructive sleep apnea (OSA) [25–28], in whom the benefit of chronotherapy has been demonstrated in some clinical trials. In a randomized, open labeled, cross over study of 41 patients with DM, bedtime administration of antihypertensive medications significantly reduced both nighttime BP (7.5 mmHg, P < 0.001) and 24 h BP (3.1 mmHg, P = 0.014) without significant difference in morning BP surge [28]. In a prospective non placebo clinical trial of 32 patients with CKD and non-dipping pattern, one antihypertensive drug was shifted from morning to evening. Results showed a decrease in the night/day mean ABP ratio in 93.7% of patients and normal circadian rhythm restored in 87.5% of patients [29]. In a meta-analysis of five randomized controlled trials of 3732 patients with CKD, bedtime dosing was associated with a 40% reduction (95% CI: 43-84%) in non-dipping patterns and significant decreases in nocturnal systolic BP (SBP: -3.17 mmHg, 95% CI: -5.41 to -0.94 mmHg) and nocturnal diastolic BP (DBP: -1.37 mmHg, 95% CI: -2.05 to – 0.69 mmHg) with a small but significant increase in awake SBP (1.15 mmHg, 95% CI: 0.10–2.19 mmHg) [30]. Similar to CKD, some data suggest that bedtime dosing might be beneficial in OSA. In a prospective, cross over trial of 41 patients with newly diagnosed hypertension and asymptomatic OSA (apnea-hypopnea index (AHI) ≥ 15/hour), patients received treatment with valsartan or valsartan/amlodipine for eight weeks in a single morning dose followed by an 8-week regimen in a single bedtime dose. Results showed further reduction of nighttime SBP/DBP by 4.4 ± 8.6/2.9 ± 5.6 mmHg (P = 0.007 and P = 0.006, respectively) and increase in dippers from 34 to 61% in the bedtime dosing group [31]. However, currently there are no data to show that reducing nocturnal hypertension by bedtime dosing could reduce cardiovascular outcomes in patients with CKD or OSA. One other important point to consider with regard to bedtime dosing is that it might be associated with a lower compliance, which might affect BP control in the long term [12, 32]. In summary, there are no consistent evidence to suggest that routine administration of antihypertensive medications at bedtime could improve nocturnal BP or early morning BP control. However, for cases with uncontrolled nocturnal hypertension and morning hypertension despite sufficient administration of long-acting antihypertensive medications in the morning, bedtime dosing might be considered.

### Evidence for the efficacy of evening dosing in reducing cardiovascular outcomes

As mentioned earlier, some studies, mainly from a single research group in Spain, have shown that bedtime dosing of one or more antihypertensive medications can reduce cardiovascular events beyond the degree of nocturnal BP lowering. The first study to demonstrate this benefit was the aforementioned MAPEC study, which demonstrated that bedtime dosing of one or more antihypertensive medications, compared to morning dosing, was associated with 61% lowering of total cardiovascular disease (CVD) events and 67% reduction in major events (CVD death, myocardial infarction, ischemic stroke and hemorrhagic stroke) [5]. In a substudy of the MAPEC study done on patients with type 2 diabetes who were randomized to either morning dosing or bedtime dosing of ≥ 1 medications with a median follow-up of 5.4 years, bedtime dosing was associated with a 67% lower risk of total cardiovascular events and a 75% lower risk of major CVD events (CVD deaths, myocardial infarction (MI) and stroke) [6]. The latest trial to show benefit of bedtime dosing was the aforementioned Hygia chronotherapy trial, which demonstrated a 45% reduction of CVD outcome, a 56% reduction of CVD death, a 34% reduction of MI, a 40% reduction of coronary revascularization, a 42% reduction of heart failure, and a 49% reduction of stroke [7]. However, the Hygia study has been under scrutiny and criticisms for many aspects, especially with regard to its design and implausible results of the study when considering the degree of BP reduction. When considering that there was approximately 4 mmHg more lowering of nocturnal BP for bedtime dosing in the Hygia study, this would mean that there was an approximately 10% reduction in cardiovascular outcomes for every 1 mmHg reduction in nocturnal BP. This is a large discrepancy compared to results reported by major cohort studies. For example, in the Dublin outcome study, there was an increase in the relative hazard ratio of 21% for every 10 mmHg increase in nighttime systolic BP [1]. The same concerns could be applied to the MAPEC study, which demonstrated a 67% reduction in cardiovascular events despite approximately 5 mmHg further reduction of nocturnal BP in the bedtime dosing arm and the MAPEC DM substudy, which demonstrated a 67% reduction in cardiovascular events with approximately 7 mmHg further reduction of nocturnal BP in the bedtime dosing arm [5, 6].

Regarding concerns for the Hygia study in the Scientific community, the European Heart Journal investigated the content and conduct of the study and declared that there was no ground for an ethical or factual concern [33]. However, the European Society of Cardiology stated that they could not verify source data and recommended that the director of the Universidade de Vigo should perform such an investigation [8]. Due to uncertainties of the clinical evidence, the International Society of Hypertension recently published a consensus document that did not endorse the use of bedtime dosing of antihypertensive medications [9].

The latest study to cast more doubt on the efficacy of bedtime dosing in reducing cardiovascular events is the TIME study. The TIME study was a prospective, pragmatic trial that randomized 21,104 hypertensive subjects (mean age: 65.1 years) of mainly white ethnicity to take their antihypertensive medications either in the morning (6–10 AM) or in the evening (8PM to 12 AM). With a median follow-up of 5.2 years, there was no significant difference in the unadjusted hazard ratio for the primary endpoint [HR: 0.95, 95% CI(0.83–1.10), P = 0.53] defined as a composite of cardiovascular mortality, non-fatal myocardial infarction, and non-fatal stroke between the two groups. There was no significant difference in secondary cardiovascular endpoint either. Another interesting finding was that the bedtime dosing group reported higher non adherence rate compared to the morning dosing group (39.0% vs. 22.5%, *P* < 0.0001). In a subgroup analysis of 7657 subjects who submitted at least one set of home BP measurements, the bedtime dosing group was associated with a significantly lower mean morning SBP (1.8 mmHg, *P* < 0.0001) but a higher evening blood pressure (1.1 mmHg, *P* < 0.0001) [12]. These results suggest that there is a minor but significant benefit of evening dosing in reducing morning BP, with a trade-off of slightly less reduction of evening BP.

With the aforementioned issues of the Hygia study and the negative results from the TIME study, it is unclear at this time whether evening dosing is beneficial in reducing cardiovascular outcomes. Another ongoing prospective randomized study, the Bedtime versus morning use of antihypertensives for cardiovascular risk reduction (BedMed), will shed further light on this issue [34]. Against these backdrops, the KSH issued the following statements with regard to chronotherapy in hypertension.

## Conclusions

### Statements from the Korean society of hypertension on chronotherapy for treating hypertension

1. Based on current evidence at hand, there is no evidence that routine administration of antihypertensive medications in the evening/bedtime is associated with better control of nocturnal blood pressure and early morning blood pressure. Therefore, routine administration of antihypertensive medications in the evening/bedtime is not recommended.
2. To control nocturnal BP and early morning BP, prescribing once daily, long-acting antihypertensive medications in the morning is recommended. However, bedtime dosing has been shown to reduce nocturnal BP in subjects with high prevalence of nocturnal hypertension/non dipping such as DM, CKD, and OSA. Therefore, for cases with nocturnal hypertension resistant to long-acting medications administered in the morning, evening dosing of one or more of the medications might be considered.
3. The evidence that evening dosing reduces cardiovascular events is not clear at this time. Until there are more evidence to support evening dosing, the KSH does not recommend evening dosing in hypertensive patients for the purpose of reducing cardiovascular events.

### Future perspective

Currently there is no definite evidence to show that routine bedtime dosing can reduce cardiovascular outcome in hypertension. Studies that show possible efficacy of chronotherapy in reducing cardiovascular events in patients with nocturnal hypertension and/or non-dipping nocturnal BP are needed in the future.

## Data Availability

Not applicable.

## Abbreviations

ABPM: Ambulatory blood pressure monitoring
BP: Blood pressure
TIME: The Treatment in Morning versus Evening
KSH: The Korean Society of Hypertension
DM: Diabetes mellitus
CKD: Chronic kidney disease
OSA: Obstructive sleep apnea
AHI: Apnea-hypopnea index
CVD: Cardiovascular disease
MI: Myocardial infarction

## References

[1] Dolan E (2005). Superiority of ambulatory over clinic blood pressure measurement in predicting mortality: the Dublin outcome study. Hypertension.

[2] Salles GF (2016). Prognostic effect of the nocturnal blood pressure fall in hypertensive patients: the ambulatory blood pressure collaboration in patients with hypertension (ABC-H) meta-analysis. Hypertension.

[3] Kario K (2020). Nighttime blood pressure phenotype and cardiovascular prognosis: practitioner-based nationwide JAMP study. Circulation.

[4] Svensson P (2001). Comparative effects of ramipril on ambulatory and office blood pressures: a HOPE substudy. Hypertension.

[5] Hermida RC (2010). Influence of circadian time of hypertension treatment on cardiovascular risk: results of the MAPEC study. Chronobiol Int.

[6] Hermida RC (2011). Influence of time of day of blood pressure-lowering treatment on cardiovascular risk in hypertensive patients with type 2 diabetes. Diabetes Care.

[7] Hermida RC (2020). Bedtime hypertension treatment improves cardiovascular risk reduction: the Hygia Chronotherapy Trial. Eur Heart J.

[8] Brunström M (2021). Missing verification of source data in hypertension research: the HYGIA PROJECT in perspective. Hypertension.

[9] Stergiou G (2022). Bedtime dosing of antihypertensive medications: systematic review and consensus statement: International Society of Hypertension position paper endorsed by World Hypertension League and European Society of Hypertension. J Hypertens.

[10] Turgeon RD (2021). Lowering nighttime blood pressure with bedtime dosing of antihypertensive medications: controversies in hypertension - con side of the argument. Hypertension.

[11] Gumz ML (2023). Toward precision medicine: circadian rhythm of blood pressure and chronotherapy for hypertension – 2021 NHLBI Workshop Report. Hypertension.

[12] Mackenzie IS (2022). Cardiovascular outcomes in adults with hypertension with evening versus morning dosing of usual antihypertensives in the UK (TIME study): a prospective, randomised, open-label, blinded-endpoint clinical trial. Lancet.

[13] El Jamal N, et al. The circadian biology of heart failure. Circ Res. 2023;132(2):223–37. 10.1161/CIRCRESAHA.122.321369 PMC9869465 36656971

[14] Lecarpentier Y, et al. Molecular mechanisms underlying the circadian rhythm of blood pressure in normotensive subjects. Curr Hypertens Rep. 2020;22(7):50. 10.1007/s11906-020-01063-z PMC7359176 32661611

[15] Goncharuk VD, et al. Neuropeptide changes in the suprachiasmatic nucleus in primary hypertension indicate functional impairment of the biological clock. J Comp Neurol. 2001;431(3):320–30. 10.1002/1096-9861(20010312)431:3<320::aid-cne1073>3.0.co;2-2 11170008

[16] Scheer FA, et al. Daily nighttime melatonin reduces blood pressure in male patients with essential hypertension. Hypertension. 2004;43(2):192–7. 10.1161/01.HYP.0000113293.15186.3b 14732734

[17] Parati G, et al. Effects of aging on 24-h dynamic baroreceptor control of heart rate in ambulant subjects. Am J Physiol. 1995;268(4 Pt 2):H1606–12. 10.1152/ajpheart.1995.268.4.H1606 7733361

[18] Grassi G, et al. Diurnal blood pressure variation and sympathetic activity. Hypertens Res. 2010;33(5):381–5. 10.1038/hr.2010.26 20203684

[19] Kimura G, Dohi Y, Fukuda M. Salt sensitivity and circadian rhythm of blood pressure: the keys to connect CKD with cardiovascular events. Hypertens Res. 2010;33(6):515–20. 10.1038/hr.2010.47 20379191

[20] Zhao P, et al. Evening versus morning dosing regimen drug therapy for hypertension. Cochrane Database Syst Rev. 2011;2011(10):Cd004184. 10.1002/14651858.CD004184.pub2 PMC9040377 21975743

[21] Zappe DH (2015). Time of administration important? Morning versus evening dosing of valsartan. J Hypertens.

[22] Poulter NR (2018). Randomized crossover trial of the impact of morning or evening dosing of antihypertensive agents on 24-hour ambulatory blood pressure. Hypertension.

[23] White WB, Lacourciere Y, Davidai G (2004). Effects of the angiotensin II receptor blockers telmisartan versus valsartan on the circadian variation of blood pressure: impact on the early morning period. Am J Hypertens.

[24] Burnier M (2020). Circadian variations in blood pressure and their implications for the administration of antihypertensive drugs: is dosing in the evening better than in the morning?. J Hypertens.

[25] Wolf J, Hering D, Narkiewicz K (2010). Non-dipping pattern of hypertension and obstructive sleep apnea syndrome. Hypertens Res.

[26] Yeghiazarians Y (2021). Obstructive sleep apnea and cardiovascular disease: a scientific statement from the American Heart Association. Circulation.

[27] Yoon M (2022). Prevalence and prognosis of refractory hypertension diagnosed using ambulatory blood pressure measurements. Hypertens Res.

[28] Rossen NB (2014). Targeting nocturnal hypertension in type 2 diabetes mellitus. Hypertension.

[29] Minutolo R (2007). Changing the timing of antihypertensive therapy to reduce nocturnal blood pressure in CKD: an 8-week uncontrolled trial. Am J Kidney Dis.

[30] Wang C (2017). Evening versus morning dosing regimen drug therapy for chronic kidney disease patients with hypertension in blood pressure patterns: a systematic review and meta-analysis. Intern Med J.

[31] Kasiakogias A (2015). Evening versus morning dosing of antihypertensive drugs in hypertensive patients with sleep apnoea: a cross-over study. J Hypertens.

[32] Mengden T (1993). The use of self-measured blood pressure determinations in assessing dynamics of drug compliance in a study with amlodipine once a day, morning versus evening. J Hypertens.

[33] Editor’s Note (2020). Eur Heart J.

[34] Garrison SR (2022). Bedtime versus morning use of antihypertensives for cardiovascular risk reduction (BedMed): protocol for a prospective, randomised, open-label, blinded end-point pragmatic trial. BMJ Open.

